# Testicular Adrenal Rest Tumors in Patients with Congenital Adrenal Hyperplasia

**DOI:** 10.4274/jcrpe.563

**Published:** 2012-06-09

**Authors:** Esra Deniz Papatya Çakır, Fatma Şentürk Mutlu, Erdal Eren, Aliye Özlem Paşa, Halil Sağlam, Ömer Tarım

**Affiliations:** 1 Uludağ University Faculty of Medicine, Department of Pediatric Endocrinology, Bursa, Turkey; 2 Uludağ University Faculty of Medicine, Department of Radiology, Bursa, Turkey; +90 532 643 94 70edpapatya@yahoo.com

**Keywords:** Testicular adrenal rest tumors, testicular microlithiasis, congenital adrenal hyperplasia

## Abstract

**Objective:** Early diagnosis and treatment of testicular adrenal rest tumors (TART) is important for gonadal functions and fertility protection in boys with congenital adrenal hyperplasia (CAH). In this descriptive study, we investigated the prevalence of TART in boys with 21-hydroxylase deficient (21OHD) CAH followed in our pediatric endocrine clinic.

**Methods:** The study group consisted of 14 male patients with a mean age of 9.6±5.1 (range: 0.8-18.3) years. Six (42.9%) of the 14 patients were diagnosed as having salt-wasting type (SW) and eight (57.1%) patients - as having the simple virilizing (SV) form of 21OHD. Mean age at diagnosis was 2.9±2.7 (range: 0.03-6.3) years. Two different radiologists performed scrotal ultrasonography. Chronological age, bone age, and anthropometric measurements were evaluated. Serum adrenocorticotropic hormone (ACTH), 17-alpha-hydroxyprogesterone (17OHP) and androstenedione levels were also evaluated in all patients during the follow-up period.

**Results:** Scrotal ultrasonography revealed bilateral TART in two patients (14.3%) and testicular microlithiasis (TM) in four patients (28.6%). One patient had both TART and TM bilaterally. During the follow-up period, the mean serum adrenocorticotropic hormone, 17OHP and androstenedione levels in the total group of patients were 130.0±179.1 pg/mL (21.7-726.5), 5.8±3.3 ng/mL (0.8-11.4) and 4.3±4.1 (0.2-11.0) ng/mL, respectively.

**Conclusions:** Microlithiasis or TART may be frequently encountered during the follow-up of patients with CAH. In order to prevent late complications including infertility, we suggest that ultrasonographic evaluations be performed yearly in all male CAH patients.

**Conflict of interest:**None declared.

## INTRODUCTION

Congenital adrenal hyperplasia (CAH) is an inherited disease caused by deficiency of certain enzymes responsible for adrenal steroid synthesis. In about 90% of all CAH cases, the deficient enzyme is 21-hydroxylase. The aim of the treatment is to replace the deficient hormones, namely, the glucocorticoids and mineralocorticoids. However, achieving a good compliance in these patients is not always easy. While excessive exposure to glucocorticoids may result in obesity, short stature, and hypertension, insufficient replacement may lead to excessive exposure to adrenal androgens and resultant problems such as precocious pubertal development and premature closure of the epiphyses. The most important problem that these patients suffer from in adult life is infertility (1).

In male CAH patients, suppression of the hypothalamic-hypophyseal-gonadal axis by excessive adrenal androgens and testicular adrenal rest tumors (TART) are reported to be the two most important causes of infertility (2,3,4).

TART were first described in 1940 as testicular masses in patients with CAH due to 21-hydroxylase deficiency (21OHD) (5). TART may cause infertility by compressing the neighboring testicular tissues, resulting in oligospermia, or by paracrine effects of steroids secreted by the tumor tissues (4,6).

The early diagnosis of these lesions, being benign in nature, and the implementation of appropriate treatment approaches are important for the protection of gonadal functions and fertility in male patients with CAH due to 21OHD. The aim of the present study was to determine the prevalence of TART in boys with CAH due to 21OHD.

## METHODS

Scrotal ultrasonography was performed in all 14 male children with CAH due to 21OHD who were being regularly followed in our pediatric endocrine unit and who were included in this descriptive study. The mean age of the patients was 9.6±5.1 (range: 0.8-18.3) years and the mean follow-up duration was 5.6±3.6 (range: 0.7-11.9) years at the time of the study. The study protocol was approved by the Ethics Committee of Uludağ University Faculty of Medicine. Informed consent was obtained from all parents and from patients above 7 years of age.

Six (42.9%) of the 14 patients were diagnosed as suffering from the salt-wasting type (SW) of 21OHD and eight (57.1%) - as having the simple virilizing (SV) form (Tables 1 and 2). Clinical diagnosis was confirmed by hormonal profiles in all patients. Mutation analyses could not be performed. We defined adequate control of CAH as having a mean serum level of 17-alpha-hydroxyprogesterone (17OHP) ≤10 ng/mL before the morning glucocorticoid dose during the follow-up period (1).

In the total group, the mean age at diagnosis was 2.9±2.7 (range: 0.03-6.3) years, being 0.1±0.2 (0.03-0.5) years in SW patients and 4.9±1.4 (2.7-6.3) years in SV patients. All patients were treated with hydrocortisone or other pharmacologically equivalent glucocorticoids. The mean hydrocortisone dose was 16.2±3.8 (12-25) mg/m2/day during the follow-up period. Nine of the patients also received fludrocortisone therapy in a dose of 0.05 to 0.2 mg/day (Table 3). Five of the patients (patients 3, 5, 9, 10 and 12) received a combination treatment of leuprolide acetate and cyproterone acetate because of central precocious puberty. In addition to these therapies, four patients (patients 5,9,10,12) also received growth hormone treatment. Patient 13 was given tamoxifen treatment for gynecomastia for one year. Anthropometric measurements, physical examination, and hormone assays were performed in all patients every three months during the follow-up period. Pubertal development was evaluated according to Tanner staging. Prader orchidometer was used for measurements of testicular volume. Bone age assessment was done according to the Greulich and Pyle method.

A spermiogram was performed in patient 3 at ages 13 and 14.5 years.

Scrotal ultrasonography was performed in supine position using a Toshiba Aplio SSA 770, Tokyo, Japan with 7-10-MHz high-frequency linear-array transducer and was evaluated by two radiologists at the same time. Testicles were assessed in at least two dimensions.

**Hormone assays**

Mean serum adrenocorticotropic hormone (ACTH), androstenedione, and 17OHP levels were calculated from measurements recorded in the patient files during the follow-up period. Serum follicle-stimulating hormone (FSH), luteinizing hormone (LH), total testosterone (TT), and free testosterone (FT) levels were measured during the study period, before or just after the scrotal ultrasonography in patients whose pubertal stage was advanced (Tanner stage ≥2 according to testicular volume).

Serum ACTH levels were measured by electrochemiluminescence immunoassay (ECLIA), Cobas e 411 analyzer using Roche kit.Serum 17OHP and FT levels were measured using enzyme-linked immunosorbent assay (ELISA) kit (DRG instruments GmbH, Germany).Serum FSH, LH, TT and androstenedione levels were measured by the chemiluminescent microparticle immunoassay (CMIA) method on an Architect i2000 analyzer using Architect/Abbott kit. 

## RESULTS

**Figure 1:**
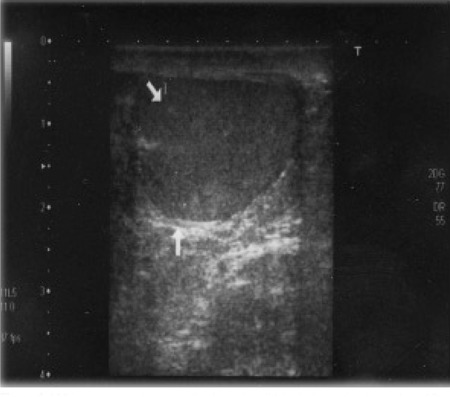
White arrows show testicular microlithiasis in patient number 13

**Figure 2:**
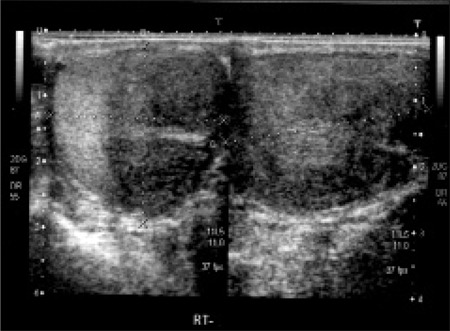
Bilateral testicular adrenal rest tumors in patient 3. The righttesticle (RT) is shown on the left and the left testicle (LT) - on the right sideof the picture. The hyperechogenic area left of the right testicle showsnormal testicular tissue compressed by hypoechogenic TART

Scrotal ultrasonography revealed bilateral TART in 2 patients (14.3%; patients 3 and 9) and testicular microlithiasis (TM) in 4 patients (28.6%; patients 9,10,12 and 13) (Figures 1 and 2). One patient (patient 9) had both TART and TM bilaterally. All patients with TART and TM had the SV form of 21OHD.

Patient 3 had large bilateral testicular tumors visualized over almost three quarters of the scrotal area (Figure 2). This patient had been followed in another pediatric endocrinology center. His age at diagnosis was 5 years. Bilateral TART were detected by scrotal ultrasonography performed at age 12 years. At this time, steroid dose was increased to a dose equivalent to 30 mg/m2/day of hydrocortisone. After one year of therapy, his TART disappeared both on scrotal ultrasonography and scrotal magnetic resonance imaging. Unfortunately, bilateral TART reappeared after decreasing the steroid dose. This patient was included in the adequate metabolic control group because his mean serum 17OHP level was 9.7 ng/mL during the follow-up period. The dimensions of TART were 29x26x35 mm in the right testicle and 29x27x35 mm in the left testicle. At thirteen years of age, his spermiogram showed one or two motile sperms in rare areas. We performed a testicular biopsy in this patient for evaluation of residual testicular tissue. The biopsy material showed adrenal rest tumors bilaterally and normal testicular tissue with mild focal dilatation in the seminiferous tubules. TART resection was not performed. This patient still receives high-dose steroid treatment.

Patient 9 had bilateral lobulated testicular masses (26x20x9.5 mm in the right and 30x14x7.5 mm in the left testicle), as well as microcalcifications in the tumoral areas. This patient’s mean serum 17OHP level was 4.7 ng/mL during the follow-up period and his metabolic control was adequate. In the above-mentioned two patients with TART, the scrotal examination revealed no palpable masses and the testis volumes were measured to be 20 mL and 25 mL, respectively. One patient (patient 12) had unilateral (left) TM, and three patients (patients 9,10 and 13) had bilateral TM (Tables 2 and 3). Patient 5 had bilateral hydrocele.

The mean serum ACTH, 17OHP and androstenedione levels in the total group of patients during the follow-up period were 130.0±179.1 pg/mL (21.7-726.5), 5.8±3.3 ng/mL (0.8-11.4) and 4.3±4.1 (0.2-11.0) ng/mL, respectively. In the patients with both TART and TM, the mean serum ACTH, 17OHP and androstenedione levels were 75.0±5.3 (27-161.8) pg/mL, 7.4±2.6 (4.7-10.3) ng/mL, and 5.2±4.8 (0.4-10.3) ng/mL, respectively during the follow-up period. The mean serum ACTH, 17OHP and androstenedione levels were 160.5±218.1 (21.7-726.5) pg/mL, 4.9±3.4 ng/mL (0.8-11.4), and 3.7±3.7 (0.2-11.0) ng/mL, respectively in the patients with neither TART nor TM. In the patients in advanced pubertal stages (testicular volume ≥ 4mL), serum FSH, LH, TT and FT levels were evaluated as well. Hormonal profile and the ultrasonographic findings of the patients are summarized in Table 2. 

## DISCUSSION

Embryological development of the adrenal cortex occurs in close proximity to the gonads. It has been suggested that adrenal rest tumors consist of adrenal tissues localized in the scrotum, within the testicles (7). In patients with CAH, TART are usually bilaterally localized, and tumor size decreases with adequate steroid replacement therapy (8). It has been reported that these tumors cause infertility in adult life via destruction of the normal testes in patients with CAH (9). On the otherhand, pathophysiology of the TM is not clearly understood; however, its association with cryptorchidism, infertility, varicocele, testicular torsion, calcification of the brain and sympathic nervous system, Down syndrome, male pseudohermaphroditism, Klinefelter syndrome, Carney complex, cystic fibrosis, germ cell tumors, and carcinoma has been reported (10,11,12,13,14,15). Poyrazoglu et al (16) recently performed scrotal ultrasonography in 41 patients with CAH and reported that 9 (21.9%) patients had TM and 9 (21.9%) had TART. Four patients in this cohort had concomitant TART and TM. To our knowledge, this study was the first report on TM in CAH patients. In our small group of patients, TART were less frequent (14.3%), but TM prevalence was slightly higher (28.6%) than that reported in Poyrazoglu’s study. One of our patients had concomitant TART and TM.

Electron microscopic appearance of TART cells resembles that of Leydig cell tumors. These tumor cells show steroid-secreting cell characteristics. However, unlike Leydig cell tumors, TART cells do not contain Reinke crystalloids (8). Val et al (17) described new cells in mice testicles and reported that these cells, visible during the embryonic period, were also found in adulthood. These new cells are called adrenal-like cells of the testis and have both adrenal and Leydig cell properties responding to both ACTH and human chorionic gonadotropin (hCG) stimulation. Although they have adrenal cell markers, these cells do not contain insulin-like factor 3 (INSL3), which is accepted to be a specific marker of Leydig cells. These investigators suggested that the hyperplasia of these cells might be responsible for developing TART. However, it has also been reported that unlike fetal mice testicles, human fetal testicles do not contain ACTH receptors. On the other hand, receptors on tumor cells are responsive to ACTH and angiotensin II. In addition, TART have also been associated with conditions showing high ACTH levels such as Nelson’s syndrome (17,18,19,20). Some authors reported that some of their well-controlled CAH patients had TART and that adequate suppression of ACTH secretion with high-dose glucocorticoid treatment was not always successful in reducing tumor size; thus they suggested the presence of growth-promoting factors other than ACTH (4,16). Our patients with TART had adequate metabolic control. However, in one patient (patient 3), TART size showed a decrease after a high-dose steroid treatment. We had only two poor metabolic control patients; one of them had TM and the other had neither TM nor TART.

The prevalence of TART in CAH patients varies between 0 and 95%, depending on tumor investigation methods, and shows an increase with age (4). Shanklin et al (21) have reported that three of seven CAH patients under eight weeks of age had TART in autopsy material. It has been reported that 15% of newborns who had inguinal exploration performed for various reasons were found to have ectopic adrenal rest tissues in their testicles and spermatic cords (22,23). Although adrenal rest tumors are predominantly seen in testicles, they may also occur in the celiac plexus, spermatic cord, liver, spinal canal, perirenal tissue, and ovaries (24,25,26,27,28,29).

TART are localized in rete testis near the testicular mediastinum and are seen as hypoechoic masses on ultrasonography (30). The majority of TART cause infertility by compressing the seminiferous tubules. It has also been hypothesized that these tumors may induce toxic paracrine effects on Leydig and/or germ cells by producing steroids (androgens and 17OHP) (30,31,32). In 2009, Claahsen-van der Grinten et al (30) suggested a staging system for TART(Table 4). According to this staging system, tumors at or higher than stage III must be surgically removed. Two of our patients had large TART in the testicular mediastinum and were classified as tumor stage III. Since one of these patients (patient 3) had large, relapsing TART bilaterally, we performed a testicular biopsy only in this patient to evaluate the residual testicular tissue and to decide whether to apply surgical resection. The biopsy of the patient showed normal testicular tissue and mild focal dilatation in seminiferous tubules. This patient is still being seen periodically.

Four of our patients had TM. The exact mechanism for formation of TM is unknown. It is speculated that degenerated seminiferous tubule epithelium covered with glycoprotein and calcium layers is responsible for the formation of TM. Prevalence of TM has been reported to be between 0.6% and 9% in the normal population (33,34,35). In our small study group, we found TM in 28.6% of patients, a rate, apparently higher than that in the normal population. TM was first reported in the autopsy material of a four-year-old healthy boy in 1970 (36). In 1987, it was sonographically visualized (14). Ultrasonography shows that TM has a hyperechogenic image. The size of the acoustic shadow differs between 1 to 3 mm and is usually seen in the testicular parenchyma, spreading peripherally or segmentally. TM is usually localized bilaterally, but may be unilateral (33). Our patients had bilateral TM except for one patient (patient 12 who had unilateral TM in his left testicle). Three of our 4 patients with TM (patients 9, 10 and 12) were treated with gonadotropin-releasing hormone (GnRH) agonists leuprolide acetate and cyproterone acetate for central precocious puberty. The mean duration of leuprolide acetate therapy was 4.05 years in these three patients with TM. One of these four patients (number 13) did not receive GnRH agonist treatment, because he did not have central precocious puberty; but he had gynecomastia and therefore tamoxifen treatment was given for one year.

We found that hormonal profile and/or level of therapeutic control of CAH did not provide a risk factor or etiologic implication for the development of TART or TM in our patients. Although TART was detected in a patient with poor compliance to treatment, resolved after higher dose of hydrocortisone and recurred after decreasing the dose, we cannot draw a conclusion from this limited observation. However, since TART cells are responsive to both ACTH and hCG, it can be speculated that adequate suppression of ACTH with appropriate glucocorticoid replacement may prevent or at least decrease the possibility of TART in patients with CAH. This possibility must be further explored in larger patient populations followed for longer periods of time.

In conclusion, we recommend yearly scrotal ultrasonographic evaluation in all male patients with CAH in order to prevent late complications including infertility. 

## Figures and Tables

**Table 1:**
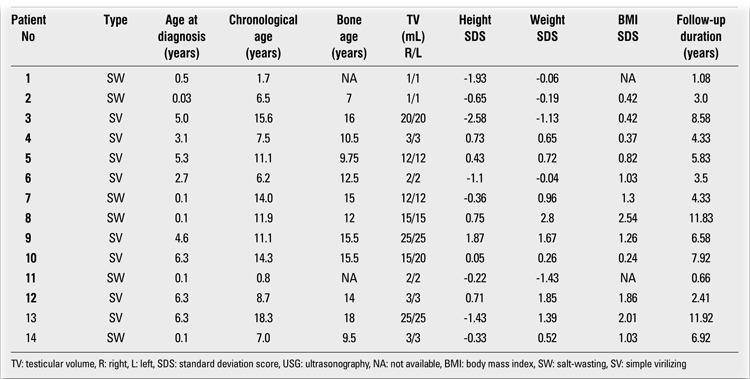
Laboratory evaluation of the patients

**Table 2:**
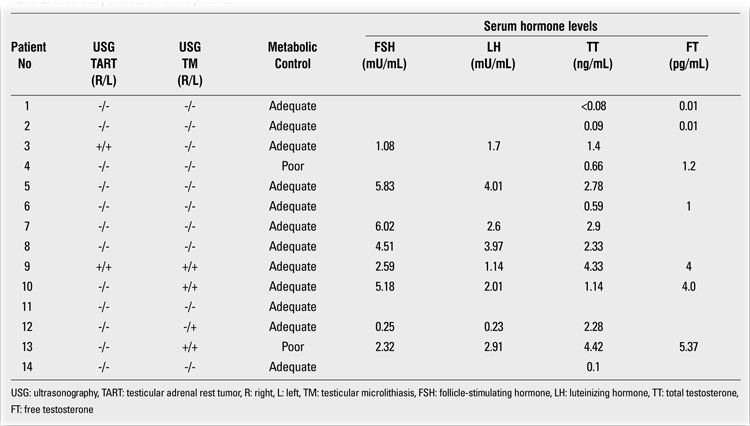
Medical treatment of the patients

**Table 3:**
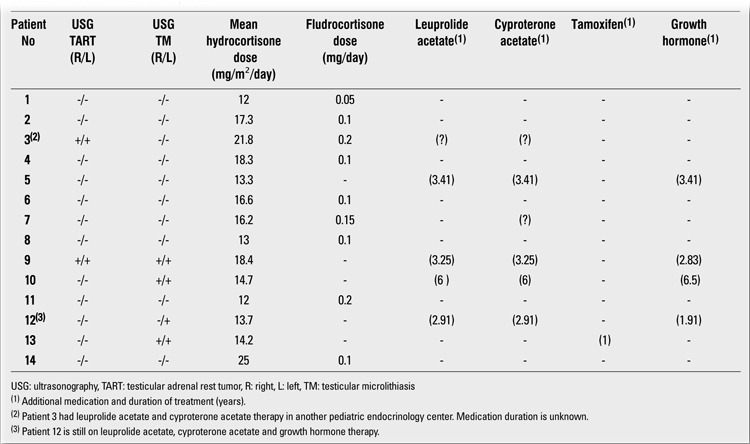
Medical treatment of the patients

**Table 4:**
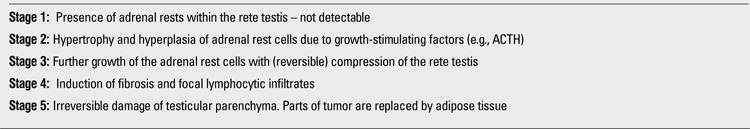
Proposed classification of testicular adrenal rests (30)
